# Injection Treatment of Hernia*Read at meeting of the East Texas Medico-Chirurgical Society, at Palestine, Texas, July 6, 1900.

**Published:** 1900-08

**Authors:** E. E. Guinn

**Affiliations:** Rusk, Texas


For the Texas Medical Journal.

### Injection Treatment of Hernia.*

BY E. E. GUINN, M. D., RUSK, TEXAS.

I shall not confine my remarks strictly to the operative treatment of hernia, but will tell you briefly what I know of the so-called injection treatment. While there are few general practitioners of medicine interested in the radical or surgical treatment of ruptures, still there is not a practicing physician who has not been or will not at some time be called upon to treat, or advise treatment for a rupture, and therefore he should be prepared to advise the best course of procedure applicable to the case. I shall not touch upon the symptoms and classifications of hernia, as this can be found in our text-books on surgery. First, I will say that all reducible hernias that can be retained by a truss, where operative or injection treatment is objected to, should be properly fitted while the patient is in the erect posture, with a truss that should be constantly worn. The truss must fit snugly and comfortably, and at all times retain the bowel, otherwise it will be worthless, absolutely harmful, and should not be worn at all. The absolute retention of the bowel is one of the essential features in the injection treatment. This treatment is especially applicable to acute hernia; I mean by "acute hernia," one that is of recent origin, where there are no adhesions between

*Read at meeting of the East Texas Medico-Chirurgical Society, at Palestine, Texas, July 6, 1900.

the sack and the surrounding tissues. With your patient lying on his back, thighs flexed, introduce your right index finger from below upward into the canal, and reduce the hernia and *sack;* then through the pubic tissues thrust your needle, guided by the index finger that is till held in position, and between tip of finger and sack, deposit ten or fifteen drops of your solution, avoiding cord, nerves and vessels; some slight pain will be experienced. Now with finger of left hand, make firm pressure over ring to prevent descent of sack and gut, remove right index finger and apply truss perfectly, and if the hernia is absolutely held in place, your patient may return to his usual duty; otherwise he should be confined to bed the whole time that he is being treated, and I think it better, in any event, that the latter course be pursued; if so, your results would be better.

From this treatment there will be a low grade of inflammation excited, as soon as this subsides (which is usually on the fourth day), the treatment should be repeated and continued four or five weeks. As a result of this treatment we expect to obliterate the canal by adhesions. A truss should be worn in most cases, perhaps for six to twelve months. Better I think than a truss, is a properly applied bandage. I believe the focused pressure produced by a truss will induce absorption. (?) The formula I have been using is—

℞ Thurja............................ʒss

Creosoti ...... ................. ♏xx

Zinci Sulph.......................gr. xv

Alcoholis ..........................ʒii

Aquæ, q.s............................℥i

All irreducible hernias should be subjected to the radical operative treatment at the earliest opportunity; delay means strangulation, sloughing and often, death. I shall describe my last operation for inguinal hernia:

Male, 23 years old, very strong.—The operation is a modification of the Bassini. After bringing together the internal oblique and transversalis, and stitching them to Poupart's ligament, the spermatic cord, which has beeen held out of the way by an assistant, is dropped into its new position just as if you were doing a Bassini operation. At this point, however, in closing the external oblique over the cord, it is done by taking up the external oblique and integument as one, and placing your sutures about one and one-half to two inches apart, packing the intervening spaces with gauze, bring about union by granulation. My object in so doing was to get a heavy and dense cicatrix completely obliterating the rings and canal, and hoping to bring about sufficient reinforcement to prevent a recurrence of the hernia, which often occurs in the Bassini operation. This operation, however, should not be attempted in septic huts, or under unfavorable circumstances, as it requires frequent dressing, thereby subjecting the wound to septic infection.

